# Oxidative Stress and Natural Antioxidants in Osteoporosis: Novel Preventive and Therapeutic Approaches

**DOI:** 10.3390/antiox12020373

**Published:** 2023-02-03

**Authors:** Gemma Marcucci, Vladana Domazetovic, Chiara Nediani, Jessica Ruzzolini, Claudio Favre, Maria Luisa Brandi

**Affiliations:** 1 Department of Experimental and Clinical Biomedical Sciences, University of Florence, 50134 Florence, Italy; 2 Department of Paediatric Haematology-Oncology, Meyer Children’s Hospital IRCCS, 50139 Florence, Italy; 3 FIRMO Foundation, Via san Gallo 123, 50129 Florence, Italy

**Keywords:** oxidative stress, natural antioxidants, inflammation, estrogen, osteoporosis, bone remodeling, osteocytes, osteoblasts, osteoclasts

## Abstract

This review reports in detail the cellular and molecular mechanisms which regulate the bone remodeling process in relation to oxidative stress (OS), inflammatory factors, and estrogen deficiency. OS is considered an important pathogenic factor of osteoporosis, inducing osteocyte apoptosis and varying levels of specific factors, such as receptor activator κB ligand (RANKL), sclerostin, and, according to recent evidence, fibroblast growth factor 23, with consequent impairment of bone remodeling and high bone resorption. Bone loss increases the risk of fragility fractures, and the most commonly used treatments are antiresorptive drugs, followed by anabolic drugs or those with a double effect. In addition, recent data show that natural antioxidants contained in the diet are efficient in preventing and reducing the negative effects of OS on bone remodeling and osteocytes through the involvement of sirtuin type 1 enzyme. Indeed, osteocytes and some of their molecular factors are considered potential biological targets on which antioxidants can act to prevent and reduce bone loss, as well as to promote bone anabolic and regenerative processes by restoring physiological bone remodeling. Several data suggest including antioxidants in novel therapeutic approaches to develop better management strategies for the prevention and treatment of osteoporosis and OS-related bone diseases. In particular, anthocyanins, as well as resveratrol, lycopene, oleuropein, some vitamins, and thiol antioxidants, could have protective and therapeutic anti-osteoporotic effects.

## 1. Introduction

Osteoporosis is a chronic systemic bone disease due to an impairment of the balance between bone formation and resorption. This disease is characterized by loss of bone mass, microarchitectural deterioration of bone tissue, and an increased risk of fractures [1]. Pain and other complications can reduce a patient’s ability to engage in activities of daily living and can be associated with significant long-term morbidity and mortality [2].

The continuous increase in life expectancy inevitably leads to an increase in diseases, especially senile osteoporosis, and the economic burden of osteoporosis-related fractures is significant [3]. Despite the numerous therapeutic approaches currently available, there has recently been an increased demand for complementary, alternative, and natural medicine including antioxidants in the prevention and treatment of osteoporosis. Understanding the effects of oxidative stress (OS) on the biological factors responsible for the development of osteoporosis and discovering the effects of natural antioxidants on the prevention and treatment of osteoporosis may lead to an improvement in the therapeutic management of this chronic disease that is so widespread worldwide [4]. Many studies have described that OS negatively impacts the bone remodeling process, causing a reduction in bone mineral density [5,6,7,8]. Recent data also show that inflammation related to OS could be part of the etiology of osteoporosis [9].

OS can be generated through several factors, such as endogenous and physiological events (metabolic alterations, hormonal changes, and aging), environmental factors (nutrition and pollution) [10,11], and pathological conditions related to inflammatory cytokines and prolonged pharmacological therapies such as corticosteroid treatments [12]. Indeed, OS is mainly due to the abnormal activation of enzymes which produce reactive oxygen species (ROS), such as superoxide anion (O_2_^−^), hydroxyl radical (OH·), and hydrogen superoxide (H_2_O_2_), the inhibition of antioxidant enzymes, and/or the reduction in exogenous and endogenous antioxidant levels [7,13]. Among the antioxidants involved in bone metabolism, the most important produced by animal cells is glutathione (GSH, γ-glutamyl-cysteinyl-glycine), a thiol antioxidant present in high concentrations within cells (2–10 mM) and able to maintain the cellular redox environment [14]. GSH is a substrate of several detoxifying enzymes, such as glutathione peroxidase (GPX) and GSH S-transferase (GST), which lead to the formation of an oxidized form of GSH (GSSG). An increase in GSSG levels induces a subsequent decrease in the GSH/GSSG ratio, and this condition is often associated with OS induction [15,16,17]. Indeed, the GSH/GSSG ratio is approximately 200:1 and is an important indicator of cellular redox state; thus, its decrease is related to a decrease in the mineralization process [15,17]. De novo GSH synthesis, GSSG reduction, and exogenous GSH uptake are essential to preserve cellular redox homeostasis. GSH is also involved in signaling pathways and is able to regulate the activity of transcription factors and intracellular proteins through reactions of glutathionylation [18,19,20].

A decrease in GSH is related to the onset of OS both in postmenopausal/senile osteoporosis and in secondary osteoporosis related to intestinal chronic disease (IBD) [7,21,22], a condition characterized by reduced intestinal absorption of antioxidants contained in food. In ovariectomized (OVX) rats, a significant increase in OS and a significant decrease in the activity of antioxidant enzymes, such as catalase, superoxide dismutase (SOD), and GPX, as well as in GSH levels, were found [20,21,22,23]; hormone replacement therapy seems to be able to reverse these effects and to maintain normal levels of nonenzymatic antioxidants, which are reduced in postmenopausal women [24]. Moreover, OS in postmenopausal osteoporosis has been related to the activation of NADPH oxidase, which produces superoxide, in association with a decreased synthesis of antioxidant enzymes and low levels of antioxidants [7,25]; hence, increased levels of hydrogen peroxide serum and reduced bone density have also been found in postmenopausal women [26,27]. Furthermore, in osteoporosis secondary to bone inflammatory processes and prolonged therapy with nonsteroidal anti-inflammatory drugs, OS is often associated with the activation of enzymes which produce ROS [28]. Recent studies have shown that, in postmenopausal osteoporosis, OS and inflammation are factors related to the imbalance of calcium–phosphorus metabolism and alteration of the mineralization process with a consequent deterioration of bone microarchitecture and increased risk of fractures [29,30,31,32,33].

The purpose of this review is to provide an overview of recent data correlating osteoporosis and alterations of the bone remodeling process to OS and proinflammatory factors at both the molecular and the cellular level. These data can better clarify the mechanisms and the possible pathogenic cofactors underlying the metabolic alterations which induce osteoporosis. An extensive review of the data on the role of natural antioxidants in counteracting bone loss and in the prevention and/or management of osteoporosis and bone inflammatory diseases related to OS is also addressed in this issue. Therefore, this review aims to describe in depth the knowledge collected in this area until now, which can lay the essential basis for future studies on the use of natural antioxidants in novel preventive and therapeutic strategies of osteoporosis.

## 2. Oxidative Stress and Proinflammatory Factors in Bone Remodeling and Mineralization Process

Several data have been described so far on the effects of OS on bone cell activity, metabolic pathways, and specific factors related to bone remodeling.

### 2.1. Oxidative Stress in Bone Remodeling and Mineralization Process

Under physiological conditions, the remodeling process is carried out by bone cells such as osteoclasts which remove damaged and old bone, osteoblasts which synthesize and secrete the osteoid matrix inducing mineralization, and osteocytes which regulate the whole process by producing specific factors. Moreover, mechanical factors (loading and exercise) and several hormones, growth factors, and cytokines are involved in the regulation of this process, essential for bone formation during the growth process and for the maintenance of healthy bone over time. Bone remodeling also allows a rapid access to calcium and phosphate for maintenance of mineral homeostasis [34]. In vivo and in vitro experiments have shown that OS affects both osteoclast and osteoblast activity, with a consequent reduction in bone mineralization and bone loss [7,17,35,36,37,38,39]. In particular, data on SaOS-2 cells, a valuable model for studying the mineralization process, report that OS induced by GSH depletion inhibits the expression and activity of alkaline phosphatase (ALP) and Runt-related transcription factor 2 (RUNX-2), both important markers related to the initial phase of osteoblast differentiation and the production of proteins, including osteocalcin, a late marker of osteoblastogenesis and essential non-collagenous protein for bone mineralization [17,35]. Moreover, OS decreases the proliferation of bone marrow mesenchymal stromal cells and osteoblast precursors [38,39]. Therefore, OS negatively affects osteoblast differentiation and activity, as well as the mineralization process. OS also correlates positively with osteoblast apoptosis and osteoclast differentiation, but negatively with osteoclast apoptosis [7,17,27]. These effects alter the bone remodeling process, causing an unbalance between osteoclast and osteoblast activity, with an increase in bone turnover rate and a scenery where resorption exceeds formation, leading to osteoporosis [9,26,29,40]. Studies on OVX rats showed that, in conditions of estrogen deficiency and increased OS, the number of osteoblasts decrease, along with their capacity to form bone and the osteoid matrix, while the number and resorptive activity of osteoclasts follow the opposite trend [41]. Indeed, in OVX rats, higher levels of markers of bone resorption (e.g., C-terminal crosslinking telopeptide type I collagen/CTX-1) and lower levels of markers of bone formation (ALP and osteocalcin) [41,42] are observed, due to an effective high turnover of the bone remodeling process in the presence of estrogen deficiency [43].

### 2.2. Role of Osteocytes in Bone Remodeling in the Presence of OS and Estrogen Deficiency

Regulation of the remodeling process is mainly related to osteocyte activity through the production of various factors, hormones, and cytokines, which regulate the differentiation and activity of osteoclasts and osteoblasts [44,45].

Osteocytes represent 90–95% of bone cells and have a long life span (up to 25 years), whereas the average lifespan of osteoblasts is 150 days [45]. Osteocytes are derived from mature osteoblasts, have a morphology similar to neuronal cells with a central body and cytoplasmic processes, and are embedded into mineralized bone tissue [46]. Osteocytes form a lacuno-canalicular system connecting them with osteoblasts, osteoclasts, bone lining cells, bone marrow cells, and blood vessels, thereby providing oxygen and nutrients for bone cells [44,45,47,48]. Moreover, osteocytes are exposed to hormones, cytokines, inflammatory factors, and signals via the bloodstream coming from other tissues and glands such as the kidney, gut, and parathyroid glands, which influence the activity of the osteocytes [45]. The interconnections among osteocytes and other bone cells are essential for regulation of the remodeling process, bone maintenance, and health.

Apoptosis of mature osteocytes represents a signal that triggers other bone cells [34,40,45,47]. Indeed, it stimulates the surrounding vital osteocytes to release signals for the induction of the lining cells to withdraw from the bone surface to form a suitable environment for the recruitment and activation of mature osteoclasts, which remove damaged bone and produce essential factors for neoangiogenesis such as vascular growth factor [34,40,47]. Osteocyte apoptosis occurs following bone matrix microdamage that induces disruption of the network; in fact, under this condition, levels of oxygen, nutrients, and factors essential for the viability of osteocytes are reduced, with consequent metabolic alterations and OS formation [49,50].

OS and osteocyte apoptosis can also occur following other physical, hormonal, and proinflammatory signals due to estrogen deficiency or an increase in factors such as TNFα, IL-6, and IL-11 in bone fluid [31,34,40]. Several data demonstrate that OS is related to osteocyte and osteoblast apoptosis [40,49,51,52], causing enhanced bone remodeling turnover and ensuing bone loss [34,40,53,54,55]. The correlation among high levels of ROS, increased OS, apoptosis of osteocytes, and alteration of bone remodeling has been confirmed by studies carried out with antioxidants. Indeed, in vivo and in vitro data demonstrate that natural antioxidants prevent and/or revert the negative effects of OS on bone tissue through the maintenance of osteocyte activity, activation of osteoblast differentiation, and the mineralization process [7,30,35,38,51]. Similarly, estrogen treatment prevents osteoblast and osteocyte apoptosis, which allows maintaining normal bone remodeling and increasing bone mineralization [9,40,55], although molecular mechanisms and biochemical signals, via which estrogens protect osteocytes from apoptosis due to microdamage and/or OS, have not been fully elucidated.

Osteocyte apoptosis seems also to have a profound role in regulating bone homeostasis in chronic inflammatory diseases, such as rheumatoid arthritis, spinal cord injury, and aging-related osteoporosis, which feature resorption-associated bone loss [56,57]. In fact, osteocyte apoptosis is correlated with elevated expression levels of proinflammatory biomarkers, including TNFα, IL-1β, IL-17, and IL-6 [28,40,58,59], which in turn are associated with increased ROS production, thus creating a toxic vicious cycle called oxinflammation [60]. In vivo studies have shown that estrogen deficiency causes a significant increase in serum TNFα, IL-6, and other inflammatory cytokine levels, and that estrogen administration improves such an inflammatory status [8,58,59,61,62].

Under physiological conditions, after the phase of bone resorption is completed, vital osteocytes also play a fundamental role in bone formation, inducing the recruitment of mesenchymal stem cells and stimulating the proliferation and differentiation of osteoblasts through activation of the Wnt/β-catenin signaling pathway and the production of osteoprotegerin (OPG) and other factors released from bone matrix [7,44,45,47].

Osteocytes contribute to the synthesis of proteins related to osteoid matrix formation, such as collagen I, osteocalcin, and osteopontin [44,45], and they produce specific proteins involved in the mineralization process and phosphate homeostasis, such as dentin matrix acidic phosphoprotein (DMP-1), sclerostin, fibroblast growth factor 23 (FGF23), matrix extracellular phosphoglycoprotein (MEPE), and phosphate-regulating neutral endopeptidase on chromosome X (PHEX) [63,64]. FGF23 is considered one of the main endocrine factors secreted by osteocytes involved in the regulation of bone remodeling and the mineralization process at the systemic and local level, together with RANKL, sclerostin, and TNFα [28,63,64,65].

Recent data have shown that, in bone and inflammatory diseases, high levels of ROS are related to increased FGF23 expression and decreased mineralization [28,31,66]. In contrast, ROS inhibition downregulated FGF23 expression in the femur of rats, which contributed to calcium and phosphate balance [67]. Recently, the upregulation of active FGF23, due to OS-induced osteocyte apoptosis and the activation of mitogen-activated protein kinases (MAPKs) with NF-κB involvement, has been demonstrated, in addition to the ability of 17β-estradiol to prevent these effects [68]. These data can be related to the ability of estrogen to downregulate osteoclastogenic factors such as sclerostin in apoptotic osteocytes [38]. In fact, it has been reported that sclerostin upregulates FGF23 expression [7,28,69], and that high levels of sclerostin inhibit bone mineralization [28,70,71]. A positive association between high serum levels of sclerostin and FGF23 has also been found in chronic kidney disease and other bone diseases related to alteration in mineral metabolism [28,70]; on the other hand, low levels of sclerostin are linked with decreased FGF23 levels, overgrowth, and sclerosis of the skeleton [69].

Several studies have demonstrated that estrogen deficiency and inflammatory diseases, including diabetes, chronic kidney disease, rheumatoid arthritis, and periodontitis, upregulate FGF23 and osteoclastogenic factors through osteocyte apoptosis and the production of inflammatory cytokines, with subsequent alteration of the osteogenic and mineralization process [28,31,40,72].

### 2.3. Pathways and Molecular Factors Involved in Redox Regulation of Bone Remodeling

Several data show that increased ROS levels are associated with an increase in apoptosis of osteocytes and/or osteoblasts, responsible for the impaired balance of bone remodeling. In particular, at the molecular level, in vitro studies have shown that increased ROS levels related to osteocyte apoptosis have mitochondrial origin, and that c-Jun-N terminal kinase (JNK) activation through mitochondrial pathways is the predominant event [51,55,73,74]. Moreover, MAPKs, such as extracellular signal-regulated kinases (ERK1/2) and p38 MAPK, are also involved in osteoblast or osteocyte apoptosis [51,73]. OS-induced apoptosis is due to the activation of caspase-3, -6, and -7, triggered by the release of cytochrome C through the outer mitochondrial membrane, thus becoming more permeable [49].

Cytochrome C-deficient mitochondria have been shown to accumulate ROS, leading to the induction of both apoptosis and osteocyte senescence [75]. The involvement of ROS and JNK in osteocyte apoptosis has been validated by in vitro studies on a murine osteocyte-like cell line, MLO-Y4. This is considered a valid model to study the signals generated by viable or apoptotic osteocytes in order to regulate the remodeling process in response to microdamage [50,75,76]. In these studies, OS-induced apoptosis in MLO-Y4 cells was obtained by starvation [51,55], an in vitro condition that mimics an in vivo metabolic state that occurs in the bone environment after microdamage and/or a lack of various endocrine factors, including estrogens [50,61,76,77]. Thiol antioxidants, such as GSH, lipoic acid (LA), and N-acetylcysteine (NAC; a cysteine analogue drug with therapeutic applications), are able to reduce ROS production and apoptosis due to caspase-3 activation; in particular, apoptosis is prevented by the specific inhibition of JNK activation [51]. Several data show that, in physiological conditions, estrogens are not direct ROS scavengers, but their antioxidant action is mostly related to the upregulation of antioxidant enzymes, such as SOD and GPX, which requires longer times [9,25,29]; therefore, the antioxidant effect of estrogens does not seem to be related to their rapid antiapoptotic action. This may also be suggested by data showing that the rapid antiapoptotic effect of estrogen is due to its ability to activate the overexpression of glutathione S-transferase P1-1 (GSTP-1), an enzyme that forms a complex with JNK and inhibits kinase activation and osteocyte apoptosis, even in the presence of an increased oxidative state [58]. Other antioxidants such as GSH, LA, and natural polyphenols can also prevent ROS increase, JNK activation, and osteocyte apoptosis [38,51,55]. These data show that both antioxidants and estrogens can rapidly prevent JNK activation and osteocyte apoptosis through regulated redox and non-redox mechanisms, suggesting a fundamental role of JNK in the regulatory activity of osteocytes during the remodeling process related to bone repair mechanisms and OS-related pathologies.

### 2.4. Relationship among RANKL/OPG Axis, Wnt/β-Catenin Signaling Pathway, and OS

Several factors produced by osteoblasts and osteocytes tightly regulate bone remodeling. Among them, RANKL, produced by both these bone cells, is one of the most important, and its binding to specific receptor RANK present on the surface of preosteoclasts activates the differentiation and activity of osteoclasts [34,40]. High levels of RANKL are also related to elevated secretion of pro-osteoclastogenic and proinflammatory biomarkers, such as TNFα, IL-6, IL-11, and HMGB1 [28], as well as intercellular cell adhesion molecule-1, VEGF-A, IGF-1, and DMP-1, all of which have an important role in bone resorption [78,79]. Studies in mouse osteocytes have shown that it is mainly RANKL produced by osteocytes, and not by osteoblasts, that activates osteoclastogenesis and is essential for normal bone remodeling [79,80]. It has also been demonstrated that ROS stimulates osteoclastogenesis through RANKL as reported in human stromal cells [26,81]. Therefore, many researchers consider osteocytes an interesting biological target for new therapeutic strategies to prevent and treat bone loss-related diseases [70,82].

Another important factor in the bone remodeling process regulation, secreted by osteocytes and osteoblasts, is OPG, a soluble receptor capable of competing with RANKL for its receptor, thus inhibiting osteoclastogenic activity and protecting the skeleton from excessive bone resorption [44]. Both RANKL and OPG are redox-regulated factors; OS upregulates RANKL expression and its release, whereas it downregulates OPG expression in osteocytes with a consequent increase in the RANKL/OPG ratio [7,28,29,51]. The increase in RANKL/OPG ratio is related to various diseases such as osteoporosis and other bone pathologies secondary to inflammation; in fact, an imbalance of this ratio is responsible for increases in bone resorption and the rate of bone remodeling turnover, not counterbalanced by an adequate and proper bone formation [29,34,45]. It has been demonstrated that the alteration of RANKL and OPG expression due to OS-induced apoptosis in osteocytes is mediated by both JNK and ERK1/2 activation [51,55]. It was also shown that activation of transcription factor NF-κB downstream of ERK1/2 signaling is related to RANKL activation, inhibition of osteoblast differentiation, and the mineralization process, whereas NF-κB activation induces osteoclast differentiation [17,35,40,83].

Apoptotic osteocytes are also able to inhibit bone formation and activate bone resorption through synthesis and release of Dickkopf protein-1 (Dkk-1) and sclerostin [7,70], inhibiting the Wnt/β-catenin signaling pathway [44,47,80]. Regarding the effects, of sclerostin it is known that sclerostin deletion in mice induces an increase in bone mineral density, whereas its overexpression decreases bone mass, and genetic mutations affecting the gene *SOST* (gene encoding sclerostin) induce sclerosteosis and van Buchem disease [44,84,85]. Indeed, sclerostin binds to LRP5/6 to inhibit Wnt signal transduction, thereby inhibiting osteoblastogenesis. In osteocytes, the increase in sclerostin expression is related to OS-induced apoptosis and activation of JNK, ERK1/2, and NF-κB signaling [17,28,40,51,86,87], with the consequent inhibition of bone formation [45,85]. The regulation of sclerostin and RANKL levels is also related to sirtuin type 1 enzyme (SIRT1); in fact, SIRT1 overexpression downregulates sclerostin in ovariectomized female mice and inhibits RANKL-induced osteoclast differentiation [86,87].

In postmenopausal women, estrogen deficiency can cause an increase in various cytokines, such as IL-1, IL-6, and TNFα, which induce an increase in RANKL, thereby stimulating osteoclastogenesis and, therefore, playing a fundamental role in the pathophysiology of osteoporosis [28,34,40]. In vivo, these events are related to estrogen deficiency, bone microdamage, and bone loss due to abnormal activation of osteoclastogenesis and alteration of the remodeling process, showing high serum levels of RANKL and sclerostin restored to normal values by estrogen therapy in postmenopausal women [7,9,88,89] Other data show that altered levels of RANKL and OPG in osteopenic and osteoporotic postmenopausal women are associated with OS markers [90]; in ovariectomized rats, OS is related to an increased expression of RANKL and osteocyte apoptosis, while estrogen treatment is able to revert these changes and preserve bone health [9]. This agrees with the ability of 17β-estradiol to prevent the increased expression of RANKL and sclerostin, as well as the high RANKL/OPG ratio, due to OS-induced apoptosis in MLO-Y4 cells via redox and non-redox mechanisms involving JNK, ERK1/2, and NF-κB signaling [55].

## 3. Natural Antioxidants and Derivatives in Bone Remodeling, the Mineralization Process, and the Treatment and Prevention of Osteoporosis

### 3.1. Natural Antioxidants and Derivatives on Bone Metabolism

Recently, many studies have explored the activity of natural antioxidants in preventing bone resorption, helping to normalize the remodeling process and contributing to bone mass formation. Several data demonstrate that natural antioxidants can prevent and/or inhibit OS, counteracting the OS-induced negative effects on molecular factors involved in bone metabolism, because they are able to inhibit factors involved in osteoclastogenesis and activate the differentiation and osteogenic activity of osteoblasts and bone matrix formation [7,30,35,38,51,91]. Natural approaches to antioxidant therapy have recently been tested in cell cultures, animals, and selected groups of patients suffering from osteoporosis or inflammatory bone diseases, highlighting their beneficial effects on bone [30,92,93]. Most of the intervention studies with antioxidants have been performed in animals, strongly suggesting a relationship between the consumption of antioxidant-rich food and bone protection, as measured by greater bone mineral content (BMC) and bone mineral density (BMD) [93].

The main natural antioxidants contained in various plants present in our diet are phenolic and nonphenolic compounds (Table 1).

### 3.2. Polyphenols

There are about 8000 polyphenols recognized in plants and fruits, about 500 of which are biologically active and obtained from natural sources, industrial food, or juices [30]. Chemically, they are phenolic substances characterized by at least two phenyl rings and one or more hydroxyl substituents. Some of these have chemical structures similar to that of 17β-E, and they may exert, in part, estrogenic activity acting through estrogen receptors [94,95,96]. Polyphenols possess antioxidant and anti-inflammatory properties, demonstrating bone-protective effects [91]. Polyphenols play an important role in the prevention and/or management of osteoporosis and bone inflammatory diseases through their ability to reduce ROS and OS [9,92,97,98,99,100]. Polyphenols, through OS inhibition, prevent precursor cells of the bone from differentiating toward cells other than those of the bone tissue [101]. They are also able to reduce osteoclast differentiation and activity, thus promoting osteoblast differentiation and, in general, favoring bone growth [95,97,100,101,102]. A daily consumption of these compounds contributes to increased bone mass peak [97,103,104], and polyphenolic antioxidant intake prevents bone loss related to increased bone fractures [92,97,102]. In fact, in a study involving 188,795 European subjects followed for 9 years, adherence to a Mediterranean diet was associated with a decreased fracture incidence [105], as well as with increased calcium absorption and retention in male adolescents [106,107].

Several studies performed in animals and in humans have shown the beneficial effects of polyphenol consumption on bone health; however, in these studies, polyphenols seemingly played a therapeutic role rather than having a protective effect [30]. Moreover, conflicting data have been reported regarding the effects of administering foods rich in polyphenols on bone disorders in postmenopausal subjects; in particular, there appear to be beneficial effects on bone health in osteopenia in postmenopausal women but not in early postmenopausal women [108,109,110,111,112,113,114,115].

In nature, polyphenols mainly exist in the form of glycosides and can be classified into flavonoids and nonflavonoids [116].

### 3.3. Flavonoids

Flavonoids are classified as reported in Table 1. The most prominent flavonoids are anthocyanins, the widespread constituents of fruits (especially blueberries and bilberries), vegetables, cereals, dry legumes, chocolate, tea, coffee, and wine. Recent data show that anthocyanins, present in blueberry as glycosides, galactosides, and arabinosides [35,117,118], are bioavailable after oral administration since they can be absorbed in intact form [94,119,120].

The effective radical-scavenging activity of flavonoids is due to the presence of a 3′,4′-dihydroxy, i.e., an o-dihydroxy group (catechol structure) in the B ring, possessing electron-donating properties and being a radical target, as well as a 3-OH group in the C ring [121]. Anthocyanins and their sugar-free counterparts, anthocyanidins, have been suggested to have beneficial effects on bone health in animals and humans [122,123,124,125,126], due to their strong antioxidant activity, contributing to the formation of bone mass [97,98,102,127,128]. In particular, flavonoids show multiple beneficial activities such as bone resorption reduction by downregulating osteoclastic activity and osteoblastic activity increase through RANKL signaling inhibition [91,129,130]. Recently, it was demonstrated that blueberry juice (BJ), rich in anthocyanins, exerts antioxidant and anti-osteoclastogenic effects in MLO-Y4 osteocytes and is able to re-establish osteogenic differentiation of SaOS-2 osteoblasts, with both cells undergoing OS, induced by starvation and glutathione depletion, respectively [35,38]. BJ is able to inhibit RANKL and sclerostin signaling through the inhibition of JNK, ERK1/2, and NF-κB activation, as well as the upregulation of the expression and activity of SIRT1, contributing to the osteogenic and mineralization process, with the involvement of ALP and RUNX-2 activation. It has also been demonstrated that blueberry polyphenols induce SIRT1 overexpression in the presence or absence of OS, in cells and in mammals [35,131]. Other data have shown that blueberry-rich diets increase bone mass density and growth by reducing RANKL activity in stromal cells, through activation of the Wnt signaling pathway [95,102].

Many authors have demonstrated that anthocyanin-rich extracts from berries are able to prevent bone loss in ovariectomy (OVX)-induced osteoporosis [98,126,128,132]; as their structure is similar to that of estrogens, they may induce their action in part through estrogen receptors and redox-independent factors related to the regulation of bone cell activity [7,38,94,95].

Moreover, blueberries are rich in *delphinidin* [133], which appears to be the most efficient anthocyanidin in preventing in vitro macrophage differentiation into osteoclasts [134,135]. Similar effects of delphinidin in medaka fish, an in vivo osteoporosis model, were reported [136]. Altogether, these data suggest that blueberry polyphenols can be useful for anabolic therapy, and that SIRT1 can be a potential target contributing to the inhibition of bone resorption and restoration of the normal remodeling process.

Phlorizidin, a polyphenol present exclusively in apples, prevents bone loss under inflammation conditions by preserving total and metaphyseal BMD and by decreasing urinary deoxypyridinoline excretion in OVX rats with and without inflammation [137]. Phlorizidin aglicone (phloretin) also displayed antioxidant activity and promoted osteoclast apoptosis in murine macrophages through JNK and p38 MAPK activation, as well as inhibited osteoporosis in OVX mice [138]. Antika et al. also showed that both these apple polyphenols are able to promote osteoblastogenesis via modulation of GSK-3β/β-catenin-dependent signaling in osteoblasts and in a mouse model of senile osteoporosis [139].

Tea catechins were investigated in the late 1990s for the presence of an ortho-dihydroxyl group in the B ring and a galloyl moiety at the third position important for their free-radical-scavenging ability [140]. Huang et al. showed that the protective role of catechins against osteoporosis was linked to their ability to restore the balance between osteoblastogenesis and osteoclastogenesis, which is inverted during osteoporosis [141].

Evidence from both animal models and in vitro experiments has suggested that green tea extract, particularly Epigallocatechin gallate, (EGCG) the most abundant green tea polyphenol, might effectively ameliorate the symptoms of osteoporosis in OVX rats and inhibit RANKL-induced osteoclast-specific gene and protein expression of cathepsin K, c-Fos, NFATc1, and c-src [142,143]. Local treatment by percutaneous injection with EGCG for 2 weeks in a rat tibial fracture model facilitated fracture healing by increasing the bone volume and improving its mechanical properties [144]; this property may be in part due to its effects on bone morphogenetic protein 2 (BMP-2), i.e., increasing its expression [145]. Interestingly, EGCG is also able to protect against glucocorticoid-induced osteoporosis in mice, by inducing β-catenin and Wnt3a protein expression, thereby increasing the transcription of the Runx-2 gene, decreasing the expression of PPAR-γ, and inducing the protein expression of cyclin D1 [146]. Wang et al. reported that EGCG protects against oxidative stress-related inhibition of osteoblastic differentiation through β-catenin and cyclin D1 expression in bone marrow-derived mesenchymal cells [147].

Vester et al. observed beneficial effects of green tea extract on mitigating oxidative stress and improving the viability of human primary osteoblasts, isolated from femoral heads of patients undergoing total hip replacement [148]. Furthermore, green tea extract in the condition of oxidative stress led to the increased expression of osteocalcin and collagen 1α1 during osteoblast differentiation [148]. In addition to green tea, black tea polyphenols, particularly theaflavin-3,3′-digallate (TFDG), were recently found through in vitro and in vivo studies [149,150,151] to display strong antioxidant and anti-inflammatory activities by reducing the levels of nitric oxide precursor, malondialdehyde (MDA), lipid peroxidation, and SOD. Furthermore, the same studies showed that serum levels of TNFα, IL-6, and RANKL were reduced, along with tartrate-resistant acid phosphatase activity, the osteoclast enzyme. A literature-based meta-analysis of 17 journal articles examining the relationship between tea intake and osteoporosis evidenced that tea consumption can significantly reduce the risk of osteoporosis in the female population but not in males, and that bad lifestyle habits may interfere negatively with this correlation [152].

Many recent clinical studies have demonstrated the association of flavonoids with increased BMD in perimenopausal and/or postmenopausal women [109,122,153]. In a 6 month study by Zhang et al. that included perimenopausal Chinese women, a combination of soy isoflavones and calcium was found to increase GSH and decrease MDA levels, without affecting SOD levels, and this independence of SOD activity was speculated to be due to the presence of genistein, a soy isoflavone with antioxidant properties [154] (Table 2).

### 3.4. Nonflavonoid Polyphenols

Phenolic acids, xanthones, tannins, and stilbenes are nonflavonoid phenolic compounds with antioxidant-related beneficial effects on bone. Phenolic acids are characterized by a carboxyl group linked to a benzene ring, whereas xanthones, stilbenes, tannins, and lignans contain at least two aromatic rings fused or linked by C–C single or double bonds.

Phenolic acids are composed of many compounds classified as hydroxybenzoic acids and hydroxycinnamic acids, some of which are worth mentioning in bone health such as curcumin and anacardic acid.

Curcumin is a lipophilic yellow polyphenol with antioxidant and anti-inflammatory properties extracted from rhizomes of the turmeric plant (*Curcuma longa*). It has been used for a long time for many diseases, including age-associated ones such as osteoporosis. It has been reported that curcumin protects against glucocorticoid-induced osteoporosis by protecting osteoblasts from apoptosis [155]. Moreover, curcumin has been shown to inhibit osteoclastogenesis by preventing the migration of bone marrow-derived macrophages and by reducing the production of CCL3, an inflammatory chemokine, as well as their subsequent differentiation to mature osteoclasts [156]. Recently, Khanizadeh et al. demonstrated that curcumin can be used complementary to alendronate to modulate BMD in postmenopausal women with osteoporosis [157].

Curcumin is also considered an autophagic activator of osteoclast precursors. Autophagy represents a crucial step in the proliferation and differentiation of osteoclasts; for this reason, the use of autophagy inhibitor drugs in association with curcumin could abrogate osteoclastogenesis, thus potentiating curcumin efficacy in treating osteoporosis [158].

Anacardic acid is a lipophilic polyphenol extracted from the shell of the cashew nut (*Anacardium occidentale*). It has been shown to exhibit several biologic activities, including antioxidant and anti-inflammatory. In particular, Zhao et al. demonstrated for the first time that treatment with anacardic acid attenuated osteoclastogenesis via the suppression of RANKL signaling in vitro and the improvement of BMD in an OVX murine model [159].

Resveratrol is the most important stilbene that can be considered a good ally for bone health. Many plants produce resveratrol in response to different forms of ambient stress, such as grapes, wine, grape juice, Itadori tea, peanuts, cocoa, blueberries, bilberries, and cranberries. Resveratrol increased bone mineral density and bone ALP in osteoporotic obese men [160], and it represented an effective therapeutic agent in preventing bone loss in ovariectomized and old rats [161]. Moreover, in OVX mice, resveratrol promoted osteogenesis by suppressing the H_2_O_2_-induced apoptosis of osteoblasts through SIRT1 upregulation and increased FoxO1 activation [162]. Shakibaei et al. demonstrated that pretreatment with resveratrol enhanced osteogenesis through the activation of SIRT1/Runx2 in mesenchymal stem cells [163]. Wang et al. also observed that resveratrol attenuated the decrease in calcium content and BMD in OVX rats by inhibiting autophagy in osteoblasts and activating it in osteoclasts [164]. Resveratrol was also found to have a protective effect on osteoporosis, induced by estrogen deficiency through the inhibition of cathepsin K expression and the Nox4/NF-kB signaling pathway [165].

Extensive evidence has suggested that the combinatory use of resveratrol and curcumin may be more effective than the individual compounds, through their synergistic inhibition of the NF-κB pathway; hence, this kind of therapeutic approach may be very useful to treat osteoarthritis and osteoporosis [166]. Yang et al. showed the protective action of resveratrol mediated by SIRT1 toward dexamethasone-induced osteoporosis in rats, whereby a relationship between increased SIRT 1 expression and bone quality was found, confirmed by in vitro models of MC3T3-E1 murine osteoblasts treated with dexamethasone [167]. The effect of resveratrol was also studied in a 24 month randomized trial in postmenopausal women, in which resveratrol administration (75 mg twice daily) showed beneficial effects on bone density in the lumbar spine and neck of the femur [168]. However, the bioavailability of resveratrol is relatively low due to its rapid metabolism and elimination, as well as the influence of the gut microbiota on its metabolism [169]. Accordingly, there is currently increasing interest in derivatives with more powerful antioxidant and anti-inflammatory properties. Some resveratrol derivatives, such as 2-phenil-benzoselenophene, were demonstrated to protect osteocytes from oxidative stress induced by starvation or GSH depletion, and the antioxidant power of these derivatives in reducing and/or restoring radical oxygen species to control values was higher than that of resveratrol itself [170].

Tannins, also known as proanthocyanidins, present in grape seed, displayed the ability to improve bone health in animal models in which bone loss was induced by ovariectomy or retinoic acid [171,172]. They also protect osteoblasts from the negative effect of glucocorticoid-induced oxidative stress through the activation of Nrf2 [173] and inhibit osteoclast formation and differentiation from bone marrow macrophages and RAW264.7 cells through the inhibition of NF-κB and JNK signaling [174].

Another polyphenol present in various fruits with remarkable protective effects against osteoporosis is ferulic acid; Hou et al. demonstrated its potential in preventing bone loss in an in vivo model of neonatal rats treated with dexamethasone to induce osteoporosis, in which the administration of ferulic acid increased BMD by activating SIRT1 and reducing NF-κB levels [175].

Moreover, extra virgin olive oil (EVOO), with all its components, especially phenolic ones, is well known for its beneficial properties, and its intake has been correlated with a reduced risk of many aging-related diseases such as cardiovascular and neurodegenerative diseases, as well as osteoporosis [176,177,178].

Hydroxytyrosol (HT), together with oleuropein (OLE) and tyrosol, is among the major polyphenolic components of EVOO; HT, in particular, represents the primary metabolite derived from the hydrolysis of OLE during the maturation and the storage of olive oil, as well as during digestion after olive oil intake. Many studies have focused on the various pharmacological activities of hydroxytyrosol, including its role in the prevention of bone loss, probably due to its antioxidant properties [179,180,181]. Interestingly, Mahmoudi et al. found a significant correlation between OLE and HT administration and an improvement of thyroid cell function, which is directly correlated with calcium homeostasis for bone health maintenance and growth [181]. In addition, treatment with OLE and HT was effective in stimulating the deposition of calcium in osteoblastic MC3T3-E1 cells, in inhibiting the formation of multinucleated osteoclasts derived from spleen cells collected from ddY mice, and in preventing bone loss in a murine model of osteoporosis [180]. Puel et al. verified that daily consumption of tyrosol and HT for 84 days improved bone loss in OVX rats [179]. The same authors showed a preventive action of bone loss by black olives in OVX rats with granulomatosis, an experimental model of senile osteoporosis, and they attributed this to the anti-inflammatory and antioxidant properties of black olives, as shown by the lower plasma fibrinogen and a1-acid glycoprotein levels and the higher levels of vitamin E [179]. Moreover, Saleh and Saleh demonstrated that EVOO intake in OVX rats was able to mitigate ovariectomy-induced osteoporosis via the prevention of hypocalcemia [182].

Other phenolic compounds in EVOO, such as caffeic acid, ferulic acid, coumaric acid, apigenin, and luteolin, when tested on MG63 osteoblasts, increased the levels of markers related to osteoblast proliferation, differentiation, and maturation, revealing their protective role in osteoporosis [183,184].

Studies in humans demonstrated that the intake of EVOO for 2 years in elderly men with high cardiovascular risk increased circulating osteocalcin, a marker of bone formation [185]. Furthermore, a trial conducted on 64 osteopenic patients, who received a polyphenol extract from *Olea europea* for 12 months daily, disclosed a significant increase in the levels of osteocalcin and a maintenance of BMD in treated participants with respect to the placebo group, where a decrease in BMD was observed [186] (Table 2).

### 3.5. Non-Polyphenolic Antioxidants

#### 3.5.1. Carotenoids

Lycopene is a lipid-soluble carotenoid acyclic isomer of beta-carotene with no vitamin A activity, mainly present in tomatoes. Its antioxidant activity has been extensively reviewed and associated with bone health benefits in postmenopausal women [187,188]. Lycopene is able to decrease osteoclast differentiation without affecting cell survival, as well as increase osteoblast proliferation and differentiation by affecting MEK signaling in both cell types, via the PKC pathway in osteoclasts and via NF-κB signaling in osteoblasts [189]. In OVX rats, femur bone remodeling and the functional activity of osteoblasts were increased following the daily intake of lycopene [190]. In a 4 month randomized study, high lycopene intake was associated with increased antioxidant capacity, decreased lipid peroxidation, and decreased protein oxidation [191]. Moreover, a dietary restriction of lycopene administration for 1 month in postmenopausal women led to a decrease in antioxidant enzyme levels and an increase in lipid and protein oxidation [190]. Recently, a pilot-controlled clinical study, conducted by Russo et al., demonstrated that the daily consumption of lycopene-rich tomato sauce containing 150 mg of lycopene prevented bone loss within a 3 month observational period [192]. An epidemiological study conducted in over 20,000 participants of middle-aged and older men and women showed that the bone density status and the risk of osteoporotic fractures were inversely correlated with dietary carotenoid intake and their plasma concentrations [193]. Moreover, the effects on bone health may differ for different carotenoids, which may be due to their antioxidant activity, as well as their different intake, as they are found in distinct concentrations in various fruits and vegetables, with differential intestinal absorption [193].

#### 3.5.2. Vitamins

Recently, some evidence has been provided on the role of vitamin C in osteoporosis development, prevention, and treatment. Brzezinska et al. performed in vitro, animal, and epidemiological studies that showed a positive effect of high vitamin C daily intake on BMD through multiple actions, including the inhibition of osteoclast activity, stimulation of osteoblast maturation, and increase in collagen type I synthesis [194]. However, the same authors reported that not all clinical studies agree with these findings, as some of them are controversial, and that a long-term observation is needed to draw an exact conclusion on this issue.

Other studies also investigated the role of vitamin E in bone health due to its antioxidant, anti-inflammatory, and immunomodulatory effects. A systematic literature research by Shuid et al. showed that vitamin E, especially tocotrienol, exerts anti-inflammatory actions in bone by diminishing IL-1, IL-6, RANKL, iNOS, and high-sensitivity C-reactive protein levels [195]. Recently, it was reported that vitamin E can have a protective effect on bone and contribute to its formation by acting at the cellular and molecular levels on RANKL and OPG levels and on the Wnt/β-catenin signaling pathway [196]. Vitamin E is able to improve bone fracture healing through an increase in the activities of antioxidant enzymes that can eliminate the excessive free radicals released during the initial phase of fracture healing [195]. Moreover, it was demonstrated that the dietary intake of vitamin E may be important to preserve and maintain bone mass in premenopausal women, correlating positively with the Z-score [197]. Shen et al. conducted a 12 week randomized single-blinded interventional study in postmenopausal women that were administered tocotrienols (TTs), a subgroup of four vitamin E isomers with strong antioxidant and anti-inflammatory properties [198]. This study showed that the supplementation of TTs significantly suppressed bone resorption biomarkers and increased bone formation biomarkers, an effect that was mediated through their antioxidant activity and the modulation of the RANKL/RANK/OPG pathway [198].

A study conducted in 405 men aged 77 to 82 years old suggested a positive correlation between BMD levels and serum vitamin E levels, confirming an association between oxidative stress and lower BMD in the male population, similarly to what was already established in women [199].

#### 3.5.3. Thiol Antioxidants

Few data have been reported on the action of thiol antioxidants on bone cell activity, although it has been demonstrated that GSH and NAC are important in preventing OS and stimulating osteoblastic differentiation in cell and animal models [13,14,17,18,51,200,201]; however, a relationship between GSH/GSSG ratio and the expression of factors involved in bone remodeling, specifically in osteogenic differentiation and the mineralization of osteoblasts, was found [17]. Indeed, it was shown that serum GSH levels decrease in postmenopausal women and OVX rats [22,23]. GSH and NAC in SaOS-2 cells were also able to increase the levels of ALP, Runx-2, and osteocalcin and decrease the RANKL/OPG ratio, thereby counteracting the negative effects of OS and inducing the activation of osteoblast differentiation and the mineralization process. In addition, they activated these osteogenic effects through mechanisms unrelated to their antioxidant activity [17], similarly to what was observed in the same cells treated with blueberry juice polyphenols in the presence or absence of OS [35]. Recently, Han et al. demonstrated that GSH attenuates RANKL-induced osteoclast formation in vitro and LPS-induced bone loss in vivo by suppressing intracellular ROS [202]. Additionally, Zhou et al. showed the protective role of NAC in preventing OVX-induced bone loss by inhibiting OS, DNA damage, cell senescence, and the senescence-associated secondary phenotype characterized by secretion of several inflammatory cytokines, chemokines, growth factors, and bioactive lipids that impact tissue remodeling [203].

The protective role of lipoic acid (LA) in bone health maintenance has been extensively examined. LA has been shown to have a protective effect on bone metabolism and osteoporosis in OVX and inflammation animal models. In fact, LA administration increased BMD levels and decreased the levels of inflammatory cytokines, osteocalcin, and osteopontin [204]. Thanks to its antioxidant and antiapoptotic activity, LA improved osteopenia by promoting the expression of markers of osteogenesis in glucocorticoid-induced osteoporosis through the regulation of NOX4, NF-κB, and PI3K/AKT signaling pathways [205]. It was also shown to inhibit TNFα- and H_2_O_2_-induced apoptosis in human bone marrow stromal cells through the mitigation of OS and prevention of JNK and NF-κB activation [206]. In addition, recent data demonstrated that GSH, NAC, and LA prevent OS and the increase in osteoclastogenic factors, such as sclerostin and RANKL, through the inhibition of JNK and ERK1/2 activity in apoptotic MLO-Y4 osteocytes [51], (Table 2).

## 4. New Potential Therapeutic Approaches for Osteoporosis: Role of Antioxidants

Depending on the severity of osteoporosis and fragility fractures, a patient’s pharmacological management is based on the choice of the most appropriate antifracture drugs, following the optimization of calcium and vitamin D levels, as well as improvements in lifestyle. Currently, the most widely used therapies for osteoporosis act mainly by inhibiting osteoclastogenesis and bone resorption in order to reduce bone loss; less commonly, therapies are used that promote bone formation or have both effects [207,208,209]. Antiresorptive therapies for osteoporosis include bisphosphonates (orally or intravenously administered) and denosumab, a monoclonal anti-RANKL antibody [209,210]. Anabolic treatments with evidence for antifracture efficacy, which enhance bone formation by increasing bone mass and strength, include teriparatide, a synthetic analogue of parathyroid hormone (1–34), [211], and abaloparatide, a synthetic analogue of PTH-related peptide (PTHrP) (not available in all countries) [212]. Lastly, a new drug for osteoporosis called romosozumab was recently approved. This new drug is a monoclonal anti-sclerostin antibody, with a dual effect of increasing bone formation and, to a lesser extent, inhibiting bone resorption [213].

The adjuvant use of antioxidants in antiresorptive therapies was recently proposed since, in inhibiting the activity of osteoclasts, they do not cause their death, which allows restoring the mutual communication between osteoclasts and osteoblasts and the physiological remodeling process, in contrast to the outcome with common antiresorptive drugs [214,215]. In fact, antioxidants act directly as ROS scavengers, inhibiting the negative effects of OS on osteogenic processes, as well as modulating the signaling pathways which activate osteocytes and osteoblasts. Accordingly, it could be interesting to design new therapeutic approaches that include the use of antioxidants to promote the restoration of anabolic processes and to develop better management strategies for the prevention of fragility fractures in primary and secondary osteoporosis; the latter is often associated with inflammatory processes and impaired oxidative metabolism [216]. Supplementation with isolated antioxidants may provide some osteoprotective benefits; however, whole plant food-derived antioxidants potentially have more overall benefits. Indeed, current evidence seems to support the fact that the antioxidant activity of nutrients is the main basis of their beneficial effects on bone metabolism, even if this is not always explicitly clear. However, larger-scale clinical trials are needed to give credence to definitive clinical recommendations.

## 5. Conclusions

This review showed that OS, together with proinflammatory factors and various interleukins, such as IL-1, IL-6, IL-11, and TNFα, constitutes one of the main factors leading to the impairment of bone metabolism and contributes to the pathogenesis of various forms of osteoporosis related to estrogen deficiency, aging, prolonged pharmacological treatments, and effects secondary to inflammatory processes.

**Figure 1:**
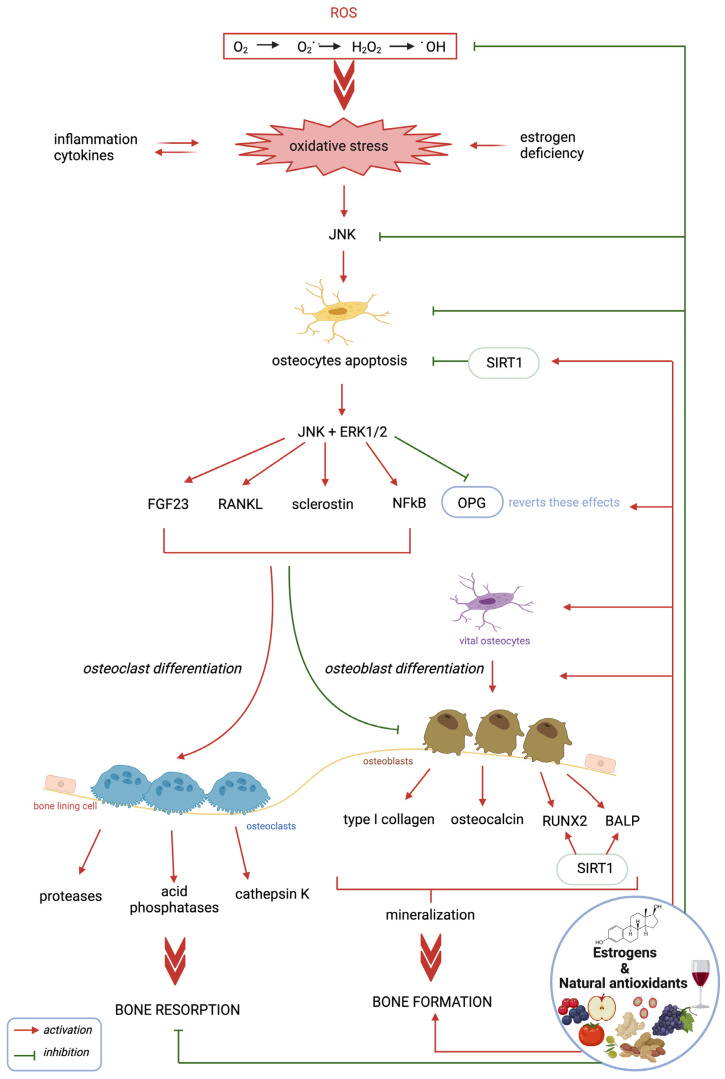
Effect of OS, antioxidants, and estrogens on osteocytes and the production of some factors involved in the bone remodeling process. OS, through JNK signaling, induces abnormal osteocyte apoptosis, which plays a central role in the regulation of specific osteocyte-related factors that induce osteoclastogenesis and bone resorption. Natural antioxidants and estrogens prevent and inhibit these effects by maintaining vital osteocytes and by regulating redox and/or non-redox intracellular mechanisms, thus favoring the process of osteogenesis and bone formation. Figure created with Biorender.com, accessed on 29 January 2023.

Several data demonstrate that bone turnover is mainly regulated at the cellular level by osteocyte activity through the specific production of hormonal and enzymatic factors, which are often redox-regulated (Figure 1).

In particular, OS-induced osteocyte apoptosis is related to increased turnover of the remodeling process and bone resorption due to osteoclast activity; conversely, a high number of vital osteocytes favors the bone formation phase through osteoblast activation, which is particularly important during bone growth and regeneration. At the molecular level, the main factors produced by apoptotic osteocytes that are responsible for excessive bone loss are RANKL, the RANKL/OPG ratio, and sclerostin, which in turn are regulated by various factors involved in signaling pathways, such as JNK, ERK1/2, and NF-κB, as well as enzymes such as SIRT1 (Figure 1). Recently, it was also shown that FGF23 is upregulated by high levels of ROS and inflammatory factors, with a consequent decrease in the osteogenic process. Therefore, many data indicate that both osteocytes and some of their specific molecular factors can be considered interesting biological targets for new drugs and therapeutic strategies to both prevent and treat OS-related bone pathologies (Figure 1).

Recent data obtained in cells, animals, and humans demonstrate that some natural antioxidants can act on these targets involved in regulation of the remodeling process, while counteracting the negative effects of OS on osteocyte activity, thereby contributing to the maintenance of an adequate activity of osteoblasts and osteoclasts. With regard to the natural antioxidants contained in various plants and fruits present in our diet, anthocyanins (polyphenols of the flavonoid group) seem to play an important role in preventing and reducing OS, excessive osteoclastogenesis, and bone loss due to OS-related diseases and inflammatory processes. These compounds, as well as other polyphenols, contribute to an increase in bone mass through redox-independent mechanisms, in part through estrogen receptors and/or the modulation of signaling pathways that activate the differentiation of bone mesenchymal stem cells, subsequently inducing bone repair and growth. Among the nonflavonoid compounds, the therapeutic effects of resveratrol and curcumin have been highlighted in postmenopausal women. Recently, tannins and ferulic acid have also shown remarkable anti-osteoporotic protective effects. Similar effects have also been found using polyphenolic compounds in EVOO, such as OLE and HT, which stimulate osteoblastic activity in particular. Among the non-polyphenolic antioxidants, lycopene affects the activity of osteoblasts, thus reducing the risk of osteoporotic fractures and promoting bone repair mechanisms. Similarly, vitamin C and vitamin E have important effects in inhibiting osteoclast activity and stimulating osteoblast activation. In particular, vitamin E is able to improve the initial phase of healing post bone fracture and to maintain bone mass in premenopausal women. Lastly, many natural antioxidants contribute to maintaining normal levels of GSH which, through its powerful antioxidant action, as well as through non-redox-regulated mechanisms, is able to promote osteogenesis and the mineralization process. Recently, among thiol antioxidants, LA was shown to be able to protect against osteoporosis and to promote the improvement of BMD levels (Table 2).

In conclusion, all reviewed studies suggest that a plant-based diet with high concentrations of particular antioxidants can improve bone mineral density, reduce the risk of age-related bone loss and osteoporosis, and promote the achievement of optimal bone mass in young people. Therefore, the use of natural antioxidants has been proposed to prevent OS bone damage, with a particular supportive role in antifracture therapies. For this purpose, further studies should be performed to clarify the molecular mechanisms underlying the activity of osteocytes and their relationship with the intracellular oxidative state, in order to identify specific targets on which antioxidants can act. Furthermore, it is necessary to thoroughly evaluate the methods of antioxidant application in alternative therapies for osteoporosis.

## Figures and Tables

**Table 1:** Natural antioxidants that may play a role in bone metabolism disorders.

Natural Antioxidants			
Polyphenols	Flavonoids	Flavonols Flavones Isoflavones Anthocyanidins Anthocyanins Flavanols Phlorizidin Epigallocatechin gallate Theaflavin-3,3′-digallate	
	Nonflavonoids	Phenolic acids	Curcumin Anacardic acid
		Xanthones	
		Tannins	
		Stilbenes	Resveratrol
		Ferulic acid	
		Hydroxytyrosol	
		Oleuropein	
		Tyrosol	
Non-polyphenolic antioxidants	Carotenoids	Lycopene	
	Vitamins	Vitamin C Vitamin E	
	Thiol antioxidants	Glutathione Alpha lipoic acid	

**Table 2:** Beneficial effects of natural antioxidants on bone metabolism.

Natural Compound	Source	Molecular Structure	Bone Effects	Ref.
Anthocyanins	Blueberries, bilberries, vegetables, cereals, dry legumes, chocolate, tea, coffee, wine	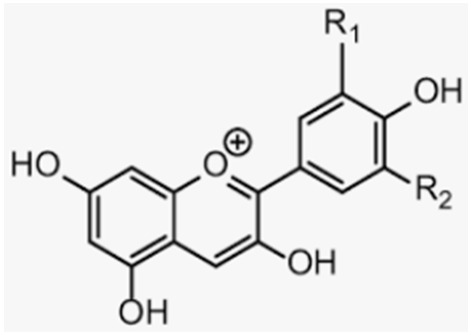	- antioxidant effect - increase in osteoblast and inhibition of osteoclast activity - prevention of bone loss in OVX-induced osteoporosis - prevention of macrophage differentiation into osteoclasts - promotion of bone growth	[35,38,91,97,98,101,127] [35,95,97,100,126] [98,126,128,129,130,132] [134,135,136] [5,97,98,102,128]
Phlorizidin	Apple	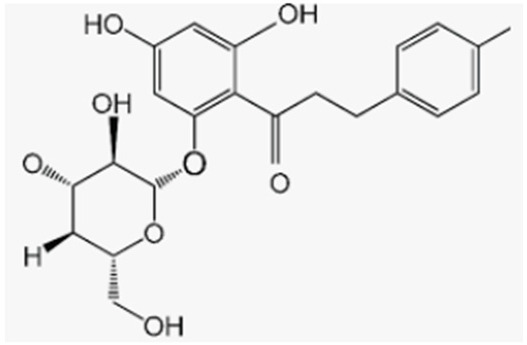	- inhibition of bone loss and preservation of total and metaphyseal BMD in OVX rats with and without inflammation	[137]
Phloretin	Apple	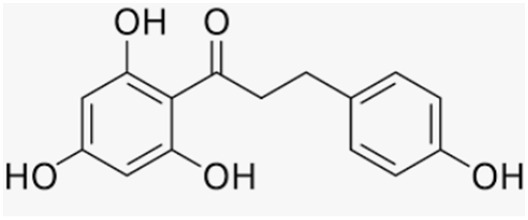	- antioxidant activity and promotion of osteoclast apoptosis in murine macrophages - promotion of osteoblast activity	[138] [139]
Epigallocatechin gallate	Green tea	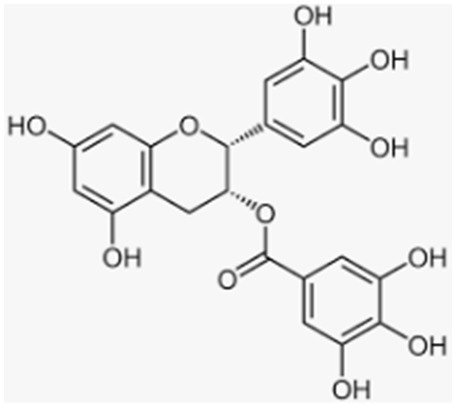	- antioxidant activity, inhibition of osteoclastogenesis, and protection from OS inhibition of osteoblast differentiation - increase in bone volume and improvement of its mechanical properties - increase in expression of osteocalcin and collagen 1α1 - protection from glucocorticoid-induced osteoporosis	[142,143,146,147,148] [144] [148] [146]
Theaflavin-3,3′-digallate	Black tea	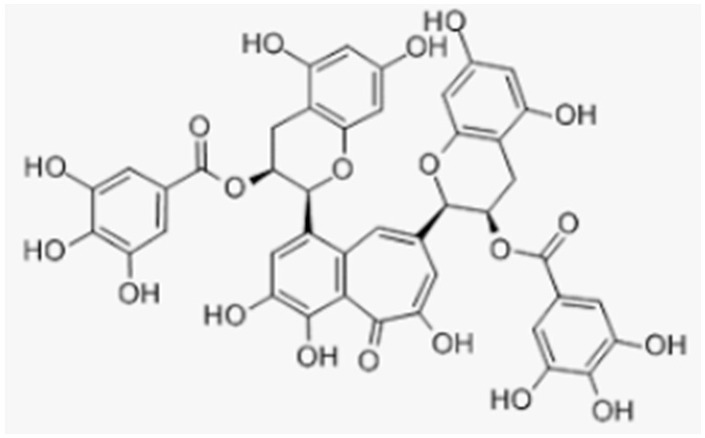	- antioxidant and anti-inflammatory action - decrease in osteoclast differentiation and promotion of the expression of osteogenic markers under an inflammatory environment	[149,151] [150,151]
Genistein	Soy	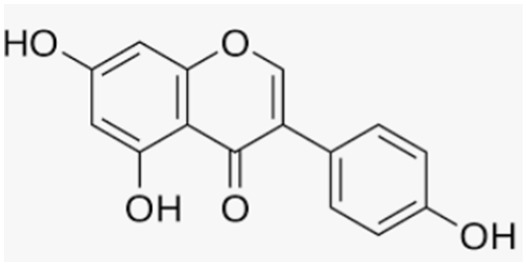	- antioxidant and anti-osteoporotic activity	[109,154]
Curcumin	Turmeric plant	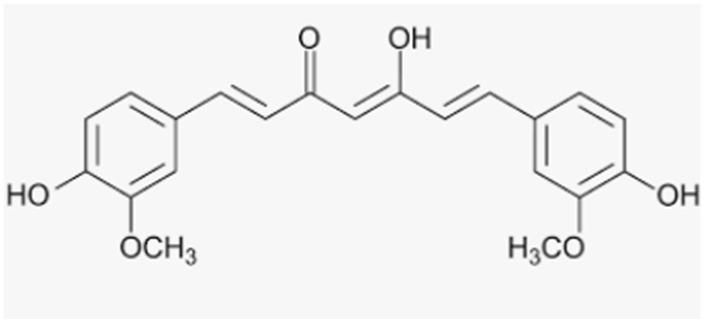	- antiapoptotic effect on osteoblasts, inhibition of osteoclastogenesis and activation of osteoclast autophagy - modulation of BMD in postmenopausal women with osteoporosis - protection from glucocorticoid-induced osteoporosis	[156,157,158] [157] [155]
Anacardic acid	Cashew nut	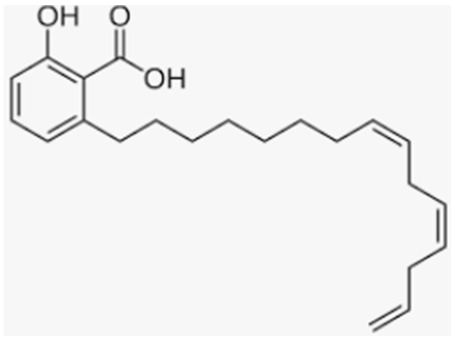	- attenuation of osteoclastogenesis and improvement of BMD in OVX murine model	[159]
Resveratrol	Grapes, wine, grape juice, Itadori tea, peanuts, cocoa, blueberries, bilberries, cranberries	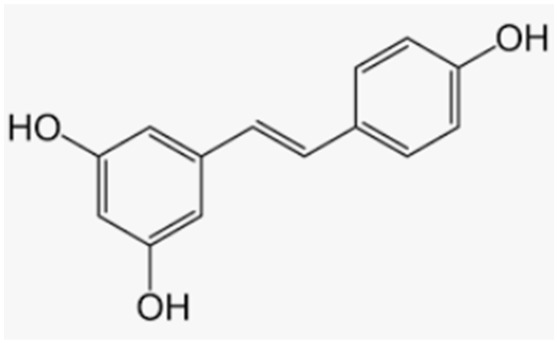	- antioxidant activity and promotion of osteogenesis - increase in bone mineral density in osteoporotic obese men - protection from estrogen deficiency and dexamethasone-induced osteoporosis in rats - beneficial effects on bone density in lumbar spine and neck of femur in postmenopausal women	[162,163,164] [160] [165,166,167] [168]
Tannins	Grape seeds	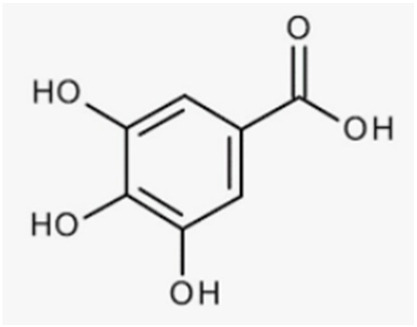	- protection of osteoblasts from glucocorticoid-induced oxidative stress - inhibition of bone marrow macrophages differentiation into osteoclasts - improvement of bone loss in ovariectomized animals	[173] [174] [171,172]
Ferulic acid	Fruits	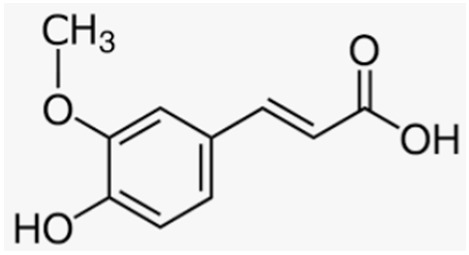	- protection from dexamethasone-induced osteoporosis	[175]
Oleuropein, hydroxytyrosol, tyrosol	Olive oil	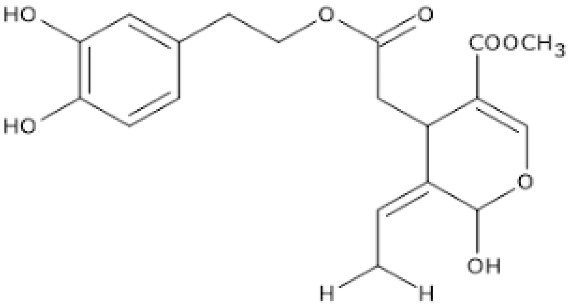	- antioxidant and anti-inflammatory effect, and prevention of bone loss - improvement of calcium homeostasis for bone health maintenance and bone growth	[179,180,181] [181,182]
Lycopene	Tomatoes	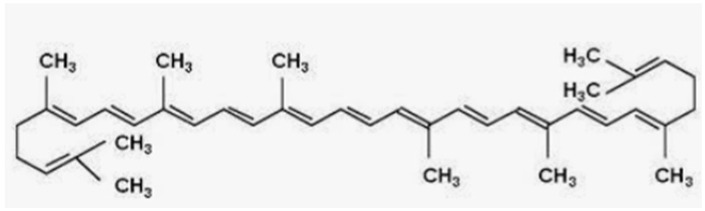	- antioxidant effect - decrease in osteoclast differentiation and increase in osteoblast activity - prevention of bone loss and reduction of osteoporotic fractures	[187,188,189,190] [191] [192,193]
Vitamin C	Fruits and vegetables	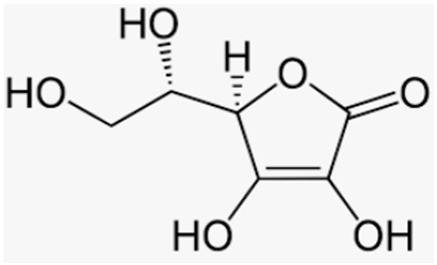	- inhibition of osteoclast activity, stimulation of osteoblast maturation, and positive effect on BMD	[194]
Vitamin E	Fruits, vegetables, seeds	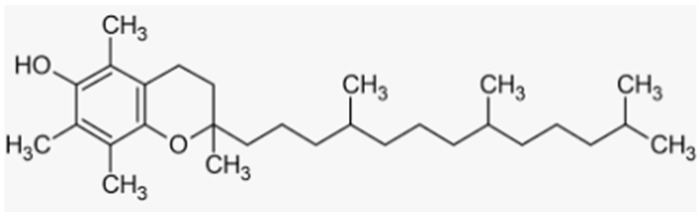	- antioxidant and anti-inflammatory effect - prevention of bone loss and induction of bone formation - preservation and maintenance of bone mass in premenopausal women	[195,198,199] [196,197,198,199] [197]
Glutathione	Intracellular	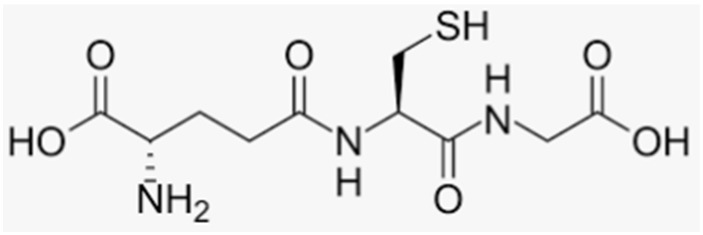	- antioxidant effect - inhibition of osteoclast activation, activation of osteoblast differentiation, and mineralization process - prevention of osteocyte apoptosis	[13,14,15,17,18] [17,202] [51]
Alpha lipoic acid	Intracellular, vegetables, red meat	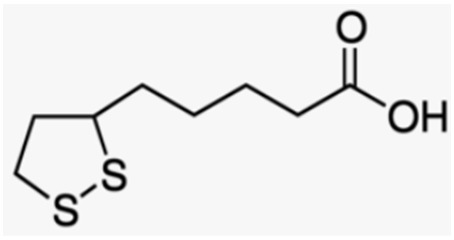	- antioxidant effect and decrease of inflammatory cytokines - prevention of osteocyte apoptosis and increase in osteoclastogenic factors - increase in BMD levels and promotion of osteogenesis in glucocorticoid-induced osteoporosis	[51,205,206] [51] [204,205]

## Data Availability

Not applicable.

## Abbreviations

oxidative stress (OS), reactive oxygen species (ROS), superoxide anion (O_2_^.−^), hydroxyl radical (OH^.^), hydrogen superoxide (H_2_O_2_), glutathione (GSH), GSH peroxidase (GPX), GSH S-transferase (GST), oxidized GSH (GSSG), superoxide dismutase (SOD), glutathione peroxidase (GPX), intestinal chronic disease (IBD), alkaline phosphatase (ALP), Runt-related transcription factor 2 (RUNX-2), mitogen-activated protein kinases (MAPKs), extracellular signal-regulated kinases (ERK1/2), c-Jun-N terminal kinase (JNK), lipoic acid (LA), N-acetylcysteine (NAC), glutathione S-transferase P1-1 (GSTP-1), ligand of receptor activator of NF-κB (RANKL), oleuropein (OLE), hydroxytyrosol (HT), osteoprotegerin (OPG), Dickkopf protein-1 (Dkk-1), sirtuin type 1 enzyme (SIRT1), fibroblast growth factor 23 (FGF23), matrix extracellular phosphoglycoprotein (MEPE), phosphate-regulating neutral endopeptidase on chromosome X (PHEX), bone morphogenetic protein 2 (BMP-2), bone mineral content (BMC), bone mineral density (BMD), malondialdehyde (MDA), theaflavin-3,3′-digallate (TFDG), nuclear factor erythroid 2-related factor (Nrf2), blueberry juice (BJ), epigallocatechin gallate, (EGCG), tocotrienols (TTs), PTH-related peptide (PTHrP), dentin matrix acidic phosphoprotein (DMP-1).
